# Non-Invasive Biomarkers and Breath Tests for Diagnosis and Monitoring of Chronic Liver Diseases

**DOI:** 10.3390/diagnostics15010068

**Published:** 2024-12-30

**Authors:** Pasawat Boon-yasidhi, Wikrom Karnsakul

**Affiliations:** 1 Department of Pharmacology, Faculty of Medicine, Chulalongkorn University, Bangkok 10330, Thailand; 2 Pediatric Liver Center, Department of Pediatric Gastroenterology, Hepatology and Nutrition, Johns Hopkins University School of Medicine, Baltimore, MD 21205, USA; wkarnsa1@jhmi.edu

**Keywords:** chronic liver disease, non-invasive test, volatile organic compound

## Abstract

**Background:** Chronic liver disease (CLD) presents a significant global health burden, demanding effective tools for diagnosis and monitoring. Traditionally, liver biopsy has been the gold standard for evaluating liver fibrosis and other chronic liver conditions. However, biopsy’s invasiveness, associated risks, and sampling variability indicate the need for reliable, noninvasive alternatives. This review examines the utility of noninvasive tests (NITs) in assessing liver disease severity, progression, and therapeutic response in patients with CLD. **Result:** Key modalities discussed include serum biomarker panels (e.g., FIB-4, APRI, ELF), imaging techniques like transient elastography, and magnetic resonance elastography, each offering unique benefits in fibrosis staging. Emerging biomarkers such as extracellular vesicles and circulating microRNAs show promise in early detection and personalized monitoring. Comparative studies indicate that while no single NIT matches biopsy precision, combinations of these modalities improve diagnostic accuracy and patient outcomes by reducing unnecessary biopsies. Moreover, NITs are instrumental in monitoring dynamic changes in liver health, allowing for more responsive and patient-centered care. **Conclusions:** Challenges remain, including standardization across tests, cost considerations, and the need for larger, diverse population studies to validate findings. Despite these limitations, NITs are increasingly integrated into clinical practice, fostering a paradigm shift toward noninvasive, accessible liver disease management. Continued advancements in NITs are essential for improved patient outcomes and will likely shape the future standard of care for CLD.

## 1. Introduction

Chronic liver disease (CLD) is a progressive condition characterized by the long-term accumulation of damage to liver parenchyma with or without liver decompensation. CLD encompasses various stages, beginning with steatosis or hepatitis and progressing through fibrosis and cirrhosis, with the potential to develop into hepatocellular carcinoma (HCC) [1]. The global prevalence of CLD is estimated at 1.5 billion cases, with metabolic dysfunction-associated steatotic liver disease (MASLD) accounting for 59%, making it the most common cause [2]. This prevalence is increasing, driven not only by reduced hepatitis virus transmission due to widespread vaccination but also by the rising rates of metabolic disorders in developed nations. As inflammation and fibrosis advance, MASLD can evolve into liver cirrhosis or HCC, both of which are associated with significantly higher morbidity and mortality [3]. This reinforces the critical need for early diagnosis and timely intervention to prevent disease progression.

Liver biopsy remains the gold standard for diagnosing and staging MASLD and cirrhosis, and it is sometimes employed for evaluating liver masses that are atypical for HCC [4]. During a transthoracic percutaneous liver biopsy, a 1–3 cm long and 1.2–2.0 mm thick tissue sample is collected using a 16–18-gauge needle, typically guided by ultrasound, aiming to retrieve 6–8 portal triads [5]. While this procedure provides definitive diagnostic information, it carries the risk of pain and complications, such as pneumothorax, hemothorax, hemobilia, sepsis, and vasovagal collapse [6]. Bleeding is the most common complication, occurring in up to 10.9% of image-guided liver biopsies, with major bleeding (requiring transfusion or surgery) affecting 0.1–4.6% of patients [7]. Liver biopsy is also contraindicated in patients who are uncooperative, have suspected vascular tumors, have a high risk of bleeding, or lack immediate access to transfusion resources [8].

Given these risks, less invasive diagnostic approaches have gained prominence. Imaging techniques like ultrasonography and vibration-controlled elastography (e.g., FibroScan^®^) can detect hepatic steatosis and cirrhosis, although their accuracy is limited in early-stage disease [9]. Magnetic resonance elastography (MRE), considered the most reliable imaging technique for fibrosis detection, offers broad liver coverage but is limited by accessibility [10]. The Fibrosis-4 (FIB-4) index, a scoring system based on age, AST, ALT, and platelet count, is commonly used to stratify MASLD risk in primary care [11]. However, it is less reliable in individuals under 35 years of age and in those with a high pretest probability of advanced disease [4]. Furthermore, these conventional methods can be labor intensive and expensive to implement, especially liver biopsy, MRE and computed tomography (CT) [12].

For cirrhosis severity assessment and prognosis, the Child–Pugh Score (CPS) and the Model for End-Stage Liver Disease (MELD) have been widely used, though both have limitations. CPS, for example, includes subjective variables such as encephalopathy and ascites grading, which are prone to inter-operator variability [13]. The MELD score, though helpful, is capped at 40, limiting its utility in more severe cases, and does not account for factors such as sodium levels or sarcopenia, leading to modified versions like MELD-Na and MELD-sarcopenia being used in some institutions [14]. Moreover, it does not account for sodium levels or sarcopenia, leading some institutions to adopt MELD-Na and MELD-sarcopenia, though these variations are not yet universally accepted. Alpha-fetoprotein (AFP) is often used for HCC prognosis and recurrence monitoring, though it is unreliable as a stand-alone diagnostic marker, as elevated AFP can result from a variety of other conditions [15].

Recent studies have proposed innovative non-invasive approaches such as volatile organic compound (VOC) analysis, metabolic breath testing, and liquid biopsy for the diagnosis and monitoring of CLD. These emerging techniques hold promise for improving diagnostic accuracy while minimizing invasiveness, representing exciting new directions for managing CLDs.

## 2. Volatile Organic Compounds

In recent years, breath biomarkers have garnered significant attention in diagnostics. VOCs are gaseous compounds produced through various metabolic processes such as carbohydrate and lipid metabolism, oxidative stress, and enzyme activity [16]. These compounds, found in exhaled breath, can be analyzed using techniques like gas chromatography, mass spectrometry, ion mobility spectrometry, and sensor arrays commonly referred to as “electronic noses” [17]. VOCs have been linked to the pathology of numerous diseases, making them a promising focus of study due to their rapid, non-invasive, and painless diagnostic potential.

Oxidative stress, mitochondrial impairment, insulin resistance, and altered metabolic pathways are hallmark features of metabolic dysfunction-associated steatohepatitis (MASH), which may be reflected in exhaled VOC levels [18]. In a pilot study involving 65 subjects, Verdam et al. identified a combination of n-tridecane, 3-methyl-butaninitrile, and 1-propanol as distinguishing MASH patients from non-MASH controls, with a sensitivity of 90% and specificity of 69% (AUROC = 0.77, 95% CI = 0.64–0.89) (Table 1) [19]. In comparison, plasma parameters such as ALT and AST showed far lower sensitivities (ALT = 19%, AST/ALT > 1 = 32%) though slightly higher specificities (AST = 96%, AST/ALT > 1 = 79%), making VOCs more suitable for screening before liver biopsy. Additionally, Sinha et al. demonstrated that specific VOCs such as isoprene (AUROC = 0.75), acetophenone (AUROC = 0.80), and terpinene (AUROC = 0.84) could differentiate non-cirrhotic MASLD patients from healthy controls [20].

The application of VOCs in children has also been explored. Alkhouri et al. studied 60 overweight children and found that mean levels of acetaldehyde, acetone, isoprene, and pentene were significantly higher in children with fatty liver compared to controls [21]. A predictive model using product ions of 14 preselected VOCs, 2-propanol, acetaldehyde, acetone, acrylonitrile, ammonia, benzene, carbon disulfide, dimethyl sulfide, ethanol, hydrogen sulfide, isoprene, pentane, triethylamine, and trimethylamine, achieved an AUROC of 0.763 for detecting MASLD. Moreover, Netzer et al. used biostatistical analysis to rank breath biomarkers with the greatest discriminatory power, demonstrating that the top-ranked VOC sets could distinguish MASLD patients from healthy individuals (AUROC = 0.96) and liver cirrhosis patients from healthy controls (AUROC = 0.94) [22,23].

Liver damage, such as cirrhosis, can result in the accumulation of metabolites in the bloodstream, which may be excreted through the breath [24]. Pijls et al. identified 11 VOCs, including 3-methylbutanal, propanoic acid, octane, terpene, terpenoid, 3-carene, branched C16H34, 1-hexadecanol, branched C16H34, dimethyl disulfide, and an unknown that were significantly different between patients with CLD and cirrhosis, achieving 83% sensitivity and 87% specificity (AUROC = 0.90) [25]. Notably, limonene, a VOC metabolized by CYP2C9 and CYP2C19, was elevated in cirrhotic patients. In comparison, serum biomarkers, including GGT, ALT, bilirubin, albumin, and thrombocytes, cumulatively achieved a sensitivity and specificity of 71% and 84% (AUROC = 0.81). In Ferrandino et al.’s study, limonene alone yielded an AUROC of 0.78 in distinguishing cirrhosis patients from controls, and machine learning models showed sensitivities ranging from 88.1% to 92.0% and specificity of 75% in detecting cirrhosis [26,27,28]. These models were also able to classify between decompensated (stage 3) and compensated (stages 1/2) with 83% sensitivity and 78% specificity.

**Table 1:** Summary of the Performance of Volatile Organic Compounds.

Study	Outcome	Participant	Volatile Organic Compound	Performance
[19]	MASH	39 overweight with MASH 26 overweight without MASH	3-VOC model: N-tridecane; 3-methyl-butanonitrile; 1-propanol.	90% sensitivity 69% specificity 0.77 AUROC
[20]	MASLD	14 MASLD 14 healthy	Isoprene	0.75 AUROC
			Acetophenone	0.80 AUROC
			Terpinene	0.84 AUROC
[21]	MASLD	37 overweight children with MASLD 23 overweight children without MASLD	14-VOC model: 2-propanol; acetaldehyde, acetone; acrylonitrile; ammonia; benzene; carbon disulfide; dimethyl sulfide; ethanol; hydrogen sulfide; isoprene; pentane; triethylamine; trimethylamine.	0.763 AUROC
[23]	MASLD	34 MASLD 35 healthy	SFR model: acetaldehyde; 5 unknown VOCs.	0.96 AUROC
	Cirrhosis	37 cirrhosis 35 healthy	SFR model: acetaldehyde; propene; 4 unknown VOCs.	0.88 AUROC
[25]	Cirrhosis	7 CLD without cirrhosis 7 CLD with cirrhosis	11-VOC model: 3-methylbutanal; propanoic acid; octane; terpene; terpenoid; 3-carene; branched C16H34; 1-hexadecanol; branched C16H34; dimethyl disulfide; 1 unknown VOC.	83% sensitivity 87% specificity 0.90 AUROC
[27]	Cirrhosis	44 cirrhosis 40 healthy	Limonene	73% sensitivity 77% sensitivity 0.78 AUROC
[28]	Cirrhosis	39 cirrhosis 11 healthy	Machine learning models	88–92% sensitivity 75% specificity 0.816–0.835 AUROC
	Decompensated Cirrhosis	10 decompensated cirrhosis 19 compensated cirrhosis		83% sensitivity 78% specificity
[29]	Cirrhosis	46 cirrhosis 42 healthy	Pentanone	0.82 AUROC
			Eucalyptol	0.80 AUROC
			Limonene	0.79 AUROC
			29-VOC model	0.95 AUROC
[30]	Cirrhosis	22 cirrhosis 32 healthy	Breath VOCs	0.965 AUROC
			Urinary VOCs	0.950 AUROC
[31]	Cirrhosis	89 cirrhosis	Breath-print analysis	2.77 (1.10–6.98) HR for mortality 2.16 (1.10–4.19) HR for hospitalization
[32]	HCC	97 HCC 111 healthy	6-VOC model: acetone; 1,4-pentadiene; phenol; methylene chloride; allyl methyl sulfide; benzene.	76.5% sensitivity 82.7 specificity
	TACE/PLAT response	22 TACE/PLAT responders 12 TACE/PLAT non-responders	Acetone dimer	77.3% sensitivity 83.3% specificity
[33]	HCC	124 HCC 210 non-HCC	Acetone dimer	83.9% sensitivity 79.4% specificity 0.816 AUROC
		124 HCC 124 cirrhosis		83.9% sensitivity 69.9% specificity 0.769 AUROC
	TACE/PLAT response	23 TACE/PLAT responders 15 TACE/PLAT non-responders		95.7% sensitivity 73.3% specificity 0.780 AUROC
	HCC	124 HCC 210 non-HCC	XGBoost algorithm: ethanol; acetone monomer; dimethyl sulfide; 1,4-pentadiene; benzene; isopropyl alcohol; acetone dimer; acetonitrile; toluene.	70.0% sensitivity 88.6% specificity
	HCC mortality	124 HCC	Isopropyl alcohol	7.23 (1.36–38.54) HR for mortality
[34]	HCC	87 early HCC 90 cirrhosis	Acetone dimer	86.2% sensitivity 87.6% specificity 0.908 AUROC
		87 early HCC 72 HBV		88.5% sensitivity 90.3% specificity 0.922 AUROC
		87 early HCC 162 non-HCC		88.5% sensitivity 82.7% specificity 0.914 AUROC

MASH: metabolic dysfunction-associated steatohepatitis, VOC: volatile organic compound; AUROC, area under receiver operating characteristic; MASLD: metabolic dysfunction-associated steatotic liver disease; SFR: Stack feature ranking; CLD: chronic liver disease; HR: hazard ratio; HCC: hepatocellular carcinoma; TACE: transarterial chemoembolization; PLAT: percutaneous local ablation therapy; XGBoost: Extreme Gradient Boosting; HBV: hepatitis B virus.

In 2023, Ferrandino et al. conducted a study on 29 VOCs collected from 46 cirrhosis patients and 42 healthy controls [29]. Eleven VOCs, eucalyptol, limonene, dimethyl selenide, 3 terpenes, 2 2-pentanones, and two unknowns, were identified to be significantly correlated with serum markers of liver function (bilirubin, albumin, and prothrombin time), showing their potential use in differentiating cirrhosis severity. Moreover, seven VOCs were selected as the best performers in detecting cirrhosis, with the top three being 2-pentanone (AUROC = 0.82), eucalyptol (AUROC = 0.80), and limonene (AURPC = 0.79), and a model constructed from their combinations was able to detect cirrhosis with an AUROC of 0.95 ± 0.04. These results were repeated by Zaim et al., where both breath and urinary VOCs excelled in differentiated cirrhosis patients from healthy controls with an AUROC of 0.965 and 0.950, respectively [30].

Further studies have examined VOCs in monitoring post-liver transplantation. Fernandez et al. reported a gradual decrease in VOCs such as limonene, methanol, and 2-pentanone following successful liver transplants in 31 cirrhotic patients, highlighting their potential role in tracking hepatic function recovery [35]. This was especially true for limonene, which demonstrated a wash-out characteristic. Additionally, De Vincentis et al. found that specific VOCs were more strongly associated with adverse outcomes in cirrhosis, with hazard ratios of 2.8 for mortality and 2.2 for hospitalization when compared to MELD (HR = 1.00 for mortality; HR = 1.1 for hospitalization) [31]. Nonetheless, CPC was shown as the strongest predictor of both mortality and hospitalization.

The role of VOCs in hepatocellular carcinoma (HCC) diagnosis has also been explored. One of the pathogenes of HCC is dysregulation of cellular metabolism, which can produce detectable changes in breath analysis. For instance, cancer cells deplete glucose supplies from neighboring cells, resulting in a shift toward the lipid metabolism pathway and subsequently upregulated acetone levels [36]. In studies by Sukaram et al., six VOCs, including acetone, 1,4-pentadince, phenol, methylene chloride, allyl methyl sulfide, and benzene, were able to differentiate HCC patients from controls with a sensitivity of 76.5% and specificity of 82.7% [32]. In a follow-up study, Sukaram et al. identified acetone dimer and ethanol as key VOCs for distinguishing between HCC, cirrhosis, and healthy individuals, with classification models showing up to 88.6% specificity [33]. Moreover, a prediction model generated by an extreme gradient boosting algorithm was able to classify participants into HCC, cirrhosis, and healthy groups with 70.0% sensitivity and 76.2% specificity. Similarly, Ain Nazir et al. constructed supervised machine learning models of VOCs that can differentiate HCC patients from healthy controls with a sensitivity and specificity of 80% [37]. Additionally, these models can differentiate between HCC and cirrhosis patients with 60–80% sensitivity and 100% specificity.

Studies suggest that VOCs could also be useful for early HCC detection. Sukaram et al. (2022) found that D-limonene detected early HCC with higher sensitivity than AFP (62.8% vs. 25.6%), though with lower specificity [32]. In a 2023 multivariate analysis, acetone dimer showed stronger predictive power than AFP for detecting early HCC (OR = 9.29 vs. OR = 1.42) [33]. Acetone dimer also achieved 83.9% sensitivity and 79.4% specificity for detecting HCC, whereas AFP showed lower sensitivity (62.4%) but perfect specificity (100%). In addition, VOCs such as isopropyl alcohol have been linked to worse survival in HCC patients, exceeding the prognostic value of AFP. Furthermore, Sukaram et al. (2024) revealed that at optimal cut-offs, acetone dimer distinguished HCC from cirrhosis, HBV, and non-HCC with sensitivities of 86.2%, 88.5%, and 88.5%, and specificities of 87.6%, 90.3%, and 82.7%, respectively. In comparison, AFP showed lower performance, with sensitivities of 61.2%, 76.5%, and 67.1%, and specificities of 66.2%, 73.2%, and 65.4%, respectively [34]. Lastly, Sukaram et al. (2022) found that a reduction in acetone levels was able to differentiate between responders and non-responders to transarterial chemoembolization (TACE) and percutaneous local ablative therapy (PLAT) with a sensitivity of 77.3% and specificity of 83.3% [32]. Other VOCs have also been linked to treatment outcomes.

Collectively, these studies indicate the potential of VOCs as valuable biomarkers for diagnosing liver diseases, detecting disease severity, and monitoring treatment outcomes. Compared to traditional serum biomarkers, VOCs demonstrate superior sensitivity and overall performance, though their specificity remains limited in certain cases. Furthermore, VOCs have the potential to reduce the cost of cancer treatment, which has long been a global healthcare burden [38]. For instance, a report on lung cancer screening demonstrated that implementing VOCs as a preliminary screening tool in high-risk groups achieved an adequate cost per quality-adjusted life year compared to a low-dose CT scan [39]. While further research is needed, VOCs show potential to be cost-effective in HCC screening, potentially lowering the radiation exposure of CT scans, as well as the treatment expenses associated with late diagnosis [40]. Their non-invasive nature, combined with increasingly sophisticated detection techniques, positions VOCs as a promising tool in clinical hepatology.

## 3. Metabolic Breath Tests

The ^13^C breath test involves labeling different substrates with stable isotope ^13^C and administering them either intravenously or orally. As these substrates are metabolized by cytochrome P-450 (CYP) enzymes—the rate-limiting step of the test—they produce ^13^CO_2_, which is then exhaled using isotope ratio mass spectrometry (IRMS) or non-dispersive isotope selective infrared spectrometry (NDIRS). The proportion of exhaled ^13^CO_2_ reflects liver function and is used to diagnose and monitor liver diseases non-invasively [41].

**Figure 1:**
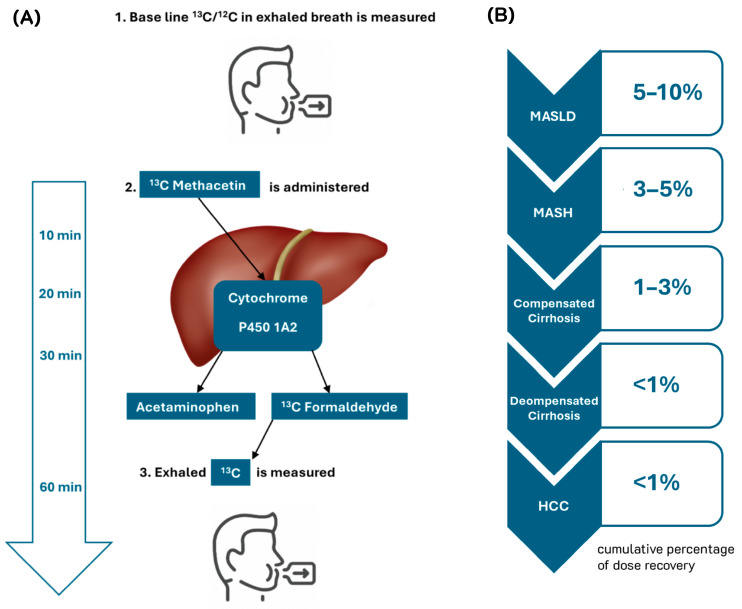
(**A-1**) In the first step of ^13^C-methacetin breath test, baseline ^13^C to ^12^C in exhaled breath is measured to establish a reference. (**A-2**) Next, the patient is administered ^13^C-labeled methacetin, which is then metabolized through cytochrome P450 1A, resulting in ^13^C-labeled formaldehyde and, eventually, ^13^C-labeled carbon dioxide. (**A-3**) Finally, the rate of ^13^C appearance in exhaled breath is measured at various intervals to determine hepatic microsomal metabolic function. (**B**) With each progressing stage of chronic liver disease, metabolic hepatic function declines. Metabolic dysfunction-associated steatotic liver disease (MASLD) involves fat accumulation without significant inflammation, so only slightly lower cumulative percentage dose of ^13^C recovery (CPDR) levels is expected. Metabolic dysfunction-associated steatohepatitis (MASH) and compensated cirrhosis involve early inflammation and fibrosis, further reducing the CPDR. Decompensated cirrhosis and hepatocellular carcinoma (HCC), often associated with end-stage liver disease, result in severely decreased CPDR.

Methacetin, rapidly metabolized by CYP1A2 in hepatocyte mitochondria into acetaminophen, serves as the basis for ^13^C-methacetin breath test [42] (Figure 1). The level of exhaled ^13^CO_2_ in this test indicates hepatic mitochondrial oxidation capacity. In a study by Fierbinteanu-Braticevici et al., cumulative ^13^C-methacetin breath test results at 60 min differentiated MASH from simple steatosis (NAFL) with a sensitivity of 95.2% and specificity of 74.4% (AUROC = 0.824) (Table 2) [43]. This test also performed well across all stages of fibrosis (AUROC = 0.824–0.973). Fibrosis in MASLD patients was detected with 73.3% sensitivity and 47.8% specificity using combined cumulative ^13^C-methacetin breath test at 40 min and time of maximum recovery, as reported by Kempinski et al., and with 65% sensitivity and 82% specificity (AUROC = 0.62) using cumulative ^13^C-methacetin breath test at 40 min alone, as reported by Razlan et al. [44,45].

Alternative breath tests have been explored for assessing hepatic function. The ^13^C-methionine breath test targets mitochondrial function through the hepatic transmethylation pathway involving sarcosine oxidase [46]. Hence, the ^13^C-methionine breath test offers another approach to evaluating mitochondrial hepatic function. Banasch et al. found that cumulative results at 90 min could distinguish MASH patients from non-MASH patients with 81% sensitivity and 76% specificity (AUROC = 0.87) [46]. The test also differentiated between advanced and mild fibrosis stages (AUROC = 0.90). Meanwhile, the ^13^C-aminopyrine breath test, metabolized by CYP2C19 via N-demethylation, reflects the liver’s microsomal metabolic capacity [56]. Tribonias et al. showed that ^13^C-aminopyrine breath test recovery at 120 min diagnosed MASH in MASLD patients with 80.0% sensitivity and 68.8% specificity (AUROC = 0.762) [47]. Galactose is another substrate utilized in the ^13^C-galactose breath test to assess cytosolic liver function [57]. Thus, ^13^C-galactose breath test is used to explore cytosolic liver function. However, Park et al. reported that the ^13^C-galactose breath test did not significantly differentiate MASH from simple steatosis in a cohort of 48 MASLD patients [47]. When Park et al. applied the ^13^C-caffeine breath test to 48 MASLD patients, it was able to distinguish MASH with a sensitivity of 79% and specificity of 80% [48].

Schneider et al. detected cirrhosis with 92.6% sensitivity and 94.1% specificity (AUROC = 0.974) using cumulative ^13^C-methacetin breath test recovery at 15 min, while Razlan et al. achieved 89% sensitivity and 83% specificity (AUROC = 0.95) with recovery at 40 min [45,49]. In a study by Stravitz et al., the cumulative dose recovery at 20 min ≤ 0.55% was chosen among other parameters to demonstrate the ability of ^13^C-methacetin breath test in cirrhosis prognosis due to its high correlation with liver-related death (HR = 12.55, 95% CI = 1.60–98.25), liver transplantation (HR = 3.19, 95% CI = 1.47–6.96), and cirrhosis complication (HR = 1.85, 95% CI = 1.08–3.17) [50]. In comparison, MELD ≥ 15, widely used as a threshold for liver transplantation, lacked the ability to prognose liver-related death (HR = 2.71, 95% CI = 0.82–8.96), while MELD ≥ 19, used in policies by United Network for Organ Sharing (UNOS) and Organ Procurement and Transplantation Network (OPTN), lacked the ability to prognose cirrhosis complication (HR = 1.67, 95% CI = 0.77–3.48). Similarly, Moran et al. found that the delta over baseline of ^13^C-methacetin breath test at 15 min ≤ 0.55% performed as well as CPS ≥ 8 and MELD ≥ 12, the optimal cut-off points of each model, in predicting mortality in decompensated cirrhosis (HR = 2.58, 95% CI = 1.17–5.69, *p* = 0.018) [51].

Jara et al. highlighted the importance of ^13^C-methacetin breath test and serum creatinine as independent predictors of 3-month liver-related mortality, leading to the creation of the CreLiMAX score, which demonstrated high predictive accuracy (AUROC = 0.83) [52]. The CreLiMAX score, derived from the combination of the two parameters, performed as well as CPS ≥ 10, MELD ≥ 16, MELD-Na ≥ 21, and United Kingdom Model for End-stage Liver Disease (UKELD) ≥ 53 in predicting 3-month mortality with sensitivity of 88% and specificity of 70% (AUROC = 0.83). Lebossé et al. reported that the ^13^C-aminopyrine breath test performed similarly in predicting the 6-month and 1-year survival of 711 cirrhosis patients, with AUROC values of 0.662 and 0.651, respectively, compared to the CPS, MELD, and indocyanine green clearance [53].

Additionally, breath tests are proving useful in monitoring liver function changes after TACE. Barzakova et al. detected short-term liver function changes in post-TACE using ^13^C-methacetin breath test, even when CPS scores remained unchanged [58]. Senk et al. observed a stronger correlation between pre-TACE ^13^C-methacetin breath test values and treatment response, as measured by Modified Response Evaluation Criteria in Solid Tumors (mRECIST) (r = 0.62, *p* < 0.05), compared to pre-TACE MELD scores (r = −0.17) [54]. Furthermore, Gairing et al. found that post-TACE ^13^C-methacetin breath test changes predicted 90-day mortality with an AUROC of 1.000, outperforming other parameters such as ALBI (AUROC = 0.952), CPS (AUROC = 0.976), and MELD (AUROC = 0.938) [55].

These metabolic breath tests are still limited by the lengthy process of isotopic ratio mass spectrometry and correlation spectroscopy, along with their high variability [59]. Exhaled CO₂ content can be altered by factors such as consuming sparkling beverages or receiving oxygen beforehand, while portosystemic shunts used in end-stage liver disease patients may reduce metabolization rates [60]. Additionally, the test results can be affected by CYP1A2 inducers such as omeprazole [61]. Currently, the only metabolic breath test for liver function approved by the U.S. Food and Drug Administration (FDA) and the European Medicines Agency (EMA) is LiMAx, which requires intravenous administration of methacetin; however, its routine use is limited due to its invasive nature [62]. Despite the limitations, metabolic breath tests have been shown to outperform conventional methods like CPS and MELD, especially regarding monitoring prognosis and treatment outcome.

## 4. Liquid Biopsies

Liquid biopsy technology has rapidly advanced, allowing for a molecular-level analysis of blood or other bodily fluids through minimally invasive means. Key components isolated from these samples include circulating tumor cells (CTCs), cell-free DNA (cfDNA), non-coding RNA (ncRNA), and extracellular vesicles (EVs). These components are analyzed using techniques such as magnetic-activated sorting, droplet digital PCR, and whole genome/exome sequencing [63].

Micro-RNA (miRNA), a class of short ncRNA, plays a critical role in the pathogenesis of MASLD. For example, miRNA-21 regulates 3-hydroxy-3-methylglutaryl-coenzyme A reductase, an enzyme overexpressed in MASLD [64]. López-Riera et al. analyzed 14 miRNAs associated with MASLD from 75 MASLD patients and 25 controls, aiming to establish diagnostic markers (Table 3) [65]. Single miRNAs detected fibrosis stages > 2 with sensitivity ranging from 72% to 83% and specificity between 60% and 74% (AUROC = 0.75–0.77), whereas the Fibrosis-4 index showed lower sensitivity (72%) but higher specificity (86%). A regression model combining miRNAs with clinical parameters further improved performance (AUROC = 0.81). Similarly, Lambrecht et al. developed the miRFIBp-score, combining circulating Platelet-Derived Growth Factor Receptor (PDGFR) and miRNAs, achieving 79.1% sensitivity and 69.5% specificity for fibrosis stage ≥ 2 (AUROC = 0.797) [66]. This scoring system detected liver fibrosis stage ≥ 2 with 79.1% sensitivity and 69.5% specificity (AUROC = 0.797, 95% CI = 0.732–0.861), outperforming other parameters, including AST/ALT, AST to platelet ratio index (PRTA), Platelet Derived Growth Factor Receptor Beta (PRTA) score, and Fibrosis-4 index. In another study by Angelini et al., a neural network classifier incorporating circulating perilipin 2, diabetes status, triglycerides, ALT, and waist circumference detected MASH with 90% sensitivity and 85% specificity (AUROC = 0.976) [67]. A logistic model incorporating monocyte RAB14 levels and metabolic parameters achieved even higher sensitivity (95.83%) and specificity (100%) for MASH detection (AUROC = 0.959), matching the performance of shear wave elastography. In comparison, ActiTest and NashTest are non-invasive diagnostic tools that utilize conventional serum biomarkers (e.g., α2-Macroglobulin, apo A1, haptoglobin, LFTs, lipid values) alongside clinical variables, offering similar sensitivity but lower specificity (20–62%) for MASH diagnosis [68]. Meanwhile, MRE demonstrates higher sensitivity (87%) and specificity (74%) but is limited by its availability.

Certain miRNAs also contribute to liver cirrhosis. For instance, transforming growth factor-beta (TGF-β) activates hepatic stellate cells, leading to fibrosis [72]. Roderburg et al. identified miR-571 and miR-652 as key players in this process as a part of the inflammatory signaling network in monocytes and macrophages, detecting cirrhosis with AUROCs of 0.91 and 0.75, respectively [69]. Combining these with miR-513-3p improved the AUROC to 0.967. A recent study by Kuang et al. further validated miRNAs as predictors of cirrhosis progression and HCC development, particularly hsa-miR-188-5p, hsa-miR-24-3p, and hsa-miR-7-5p [73].

CTCs, shed from tumors into the bloodstream, serve as tumor markers [74]. A meta-analysis by Cui et al. demonstrated that CTCs can diagnose HCC with 60% sensitivity and 95% specificity, also correlating with recurrence risk [70]. However, CTCs are best suited for predicting treatment response and recurrence due to variations in origin and release conditions [75]. Similarly, cfDNAs are derived from cell apoptosis. The fraction of cfDNA that originates from tumor cells is called circulating tumor DNA (ctDNA) and has been used for molecular diagnosis of cancer [76]. Yinzhong et al. reported that cfDNA can detect HCC with 83% sensitivity and 90% specificity (AUROC = 0.93) [71]. Additionally, ctDNA is increasingly employed to monitor chemotherapy and radiotherapy efficacy, as well as for selecting patients for targeted therapies, as miRNAs are involved in the regulation of tumor cells and have been found to be associated with recurrence and metastasis [77,78].

miRNAs have also been extensively studied as diagnostic biomarkers for HCC. A review by Shetti et al. found that miRNA sensitivity and specificity for HCC ranged from 54–96% and 60–100%, respectively (AUROC = 0.74–0.97) [79]. These miRNAs are also used to assess prognosis and treatment outcomes, particularly after TACE and radiofrequency ablation [80]. Similarly, EVs, which carry proteins, cfDNA, and ncRNAs, have shown great promise in liquid biopsy applications. Chen et al. demonstrated that EVs outperformed AFP in HCC detection, with their combination yielding even higher AUROC values [81]. Sorop et al. reinforced these findings, with exosomal miRNAs showing AUROC values between 0.702 and 0.968, with higher levels correlating with larger tumors and poorer survival outcomes [82].

Existing economic evaluations indicate that liquid biopsies are cost-effective, particularly for screening and treatment selection, though their scope is limited due to the small number of studies explicitly addressing costs [83]. The budgetary impact of using liquid biopsy HCC has yet to be thoroughly explored.

## 5. Concluding Remarks

This review demonstrates that VOC analysis holds promising accuracy for diagnosing and staging CLDs, occasionally matching or even surpassing the performance of conventional diagnostic methods. However, the underlying biological processes generating VOCs and their connection to the pathophysiology of specific diseases remain poorly understood. A recurring theme in the literature is the need to identify optimal VOC combinations with the greatest diagnostic utility for each CLD. Given the vast number of compounds detectable in human breath, future studies should incorporate machine learning into data analysis to develop standardized models with universal applicability.

Compared to VOC analysis, metabolic breath tests generally exhibit lower specificity for diagnosing CLDs. However, their utility in assessing hepatic function, prognosis, and treatment response is well-researched. Particularly, an algorithm using ^13^C-methacetin breath test over time could potentially differentiate stages of CLD independently of CPS and MELD scores. The variable time delay between substrate administration and breath analysis, ranging from minutes to hours, may limit their feasibility in outpatient settings. Addressing the challenge of optimal test timing will be crucial for increasing their practicality.

Among the non-invasive techniques reviewed, liquid biopsy emerges as the most extensively researched NIT, especially in HCC. The biological mechanisms of liquid biopsy directly correlate with tumor cell pathophysiology, and this approach has demonstrated strong diagnostic accuracy. Future research could focus on refining the selection of biomarkers, particularly the various miRNAs detectable through liquid biopsy, to enhance their diagnostic power.

In summary, these emerging non-invasive methods show significant promise. However, a lack of consistency across different NITs can affect their reliability. Future research should prioritize validating biological plausibility, standardizing study design, and evaluating cost-effectiveness before they can be standardized for routine clinical use. If implemented effectively, these tests have the potential to spare CLD patients from the complications of invasive procedures and delays in diagnosis.

## Figures and Tables

**Table 2:** Summary of the Performance of Metabolic Breath Tests.

Study	Outcome	Participant	Metabolic Breath Test	Performance
[43]	MASH	43 MASH 21 simple steatosis	^13^C-methacetin breath test (dose at 10 min)	95.2% sensitivity 76.7% specificity 0.822 AUROC
			^13^C-methacetin breath test (cumulative dose at 60 min)	95.2% sensitivity 74.4% specificity 0.824 AUROC
[44]	Fibrosis	15 MASLD with fibrosis 18 MASLD without fibrosis	^13^C-methacetin breath test (cumulative dose at 10 min)	69.2% sensitivity 47.4% specificity
			^13^C-methacetin breath test (cumulative dose at 40 min/ time of maximum level)	73.3% sensitivity 47.8% specificity
[45]	Fibrosis	21 MASLD with fibrosis 9 MASLD without fibrosis	^13^C-methacetin breath test (cumulative dose at 40 min)	65% sensitivity 82% specificity 0.62 AUROC
	Cirrhosis	30 MASLD with cirrhosis 47 MASLD without cirrhosis		89% sensitivity 83% specificity 0.95 AUROC
[46]	MASH	53 MASH 58 simple steatosis	^13^C-methionine breath test (cumulative dose at 90 min)	81% sensitivity 76% specificity 0.87 AUROC
	F2-F3 fibrosis	30 MASLD with F2-F3 fibrosis 70 MASLD with F0-F1 fibrosis		0.90 AUROC
[47]	MASH	20 MASH 16 simple steatosis	^13^C-aminopyrine breath test (dose at 120 min)	80.0% sensitivity 68.8% specificity 0.762 AUROC
			^13^C-aminopyrine breath test (cumulative dose at 120 min)	75.0% sensitivity 68.8% specificity 0.741 AUROC
[48]	F2-F4 fibrosis	23 MASLD with F2-F4 fibrosis 25 MASLD with F0-F1 fibrosis	^13^C-caffeine breath test (dose at 60 min)	0.74 AUROC
	MASH	33 MASH 15 simple steatosis		79% sensitivity 80% specificity 0.80 AUROC
	Cirrhosis	10 MASLD with cirrhosis 38 MASLD without cirrhosis		90% sensitivity 76% specificity 0.86 AUROC
[49]	Cirrhosis	35 cirrhosis 65 healthy	^13^C-methacetin breath test (cumulative dose at 15 min)	92.6% sensitivity 94.1% specificity 0.954 AUROC
[50]	Cirrhosis complication, LT, mortality	165 cirrhosis	^13^C-methionine breath test (cumulative dose at 20 min)	1.85 (1.08–3.17) HR for complication of cirrhosis 3.19 (1.47–6.96) HR for liver transplantation 12.55 (1.60–98.25) HR for liver-related mortality
[51]	Decompensated cirrhosis mortality	123 decompensated cirrhosis	^13^C-methionine breath test (delta dose at 15 min)	2.58 (1.17–5.69) HR for mortality
[52]	Cirrhosis 3-month survival	27 cirrhosis: survival ≤ 3 months 241 cirrhosis: survival > 3 months	^13^C-methionine breath test (dose at 20 min)	64% sensitivity 81% specificity 0.75 AUROC
			CreLiMAx (^13^C-methionine breath test + serum Cr)	88%sensitivity 70% specificity 0.83 AUROC
[53]	Cirrhosis 6-month survival without LT	434 cirrhosis: survival or LT ≤ 6 months 277 cirrhosis: survival or LT > 6 months	^13^C-aminopyrine breath test (dose at 60 min)	0.662 AUROC
	Cirrhosis 1-year survival without LT	545 cirrhosis: survival or LT ≤ 1 year 154 cirrhosis: survival or LT > 1 year		0.651 AUROC
[54]	TACE response	16 HCC treated with TACE	pre-TACE ^13^C-methionine breath test (dose at 20 min)	r = 0.62, *p* < 0.05 correlation with treatment response
[55]	TACE response	20 HCC treated with TACE	pre/post-TACE delta ^13^C-methionine breath test (dose at 45 min)	1.000 AUROC

MASH: metabolic dysfunction-associated steatohepatitis; AUROC: area under receiver operating characteristic; MASLD: metabolic dysfunction–associated steatotic liver disease; LT: liver transplantation; HR: hazard ratio; Cr: creatinine; TACE: transarterial chemoembolization; HCC: hepatocellular carcinoma.

**Table 3:** Summary of the Performance of Liquid Biopsies.

Study	Outcome	Participant	Liquid Biomarker	Performance
[65]	MASLD	29 MASLD with F3-F4 fibrosis 46 MASLD with F0-F2 fibrosis	miR27b/197	83% sensitivity 60% specificity 0.75 AUROC
			miR27b/30c	72% sensitivity 74% specificity 0.77 AUROC
			PLS-DA model (multiple mRNAs)	69% sensitivity 76% specificity 0.81 AUROC
			PLS-DA model + clinical variables	72% sensitivity 83% specificity 0.83 AUROC
[66]	F2-F4 fibrosis	116 MASLD with F2-F4 fibrosis 92 MASLD with F0-F1 fibrosis	miRFIB score: Let-7f-5p; miRNA-122-5p; miRNA-142-5p; miRNA-29a-3p; miRNA-451a.	78.4% sensitivity 62.4% specificity 0.756 AUROC
			miRFIBp score: miRFIB score + PDGFRβ	79.1% sensitivity 69.5% specificity 0.797 AUROC
[67]	MASH	100 MASH 50 simple steatosis	Neuronal network: perilipin 2; DM status; TG; ALT; waist circumference.	88% sensitivity 100% specificity 0.976 AUROC
			RAB14	82.4% sensitivity 96.9% specificity
			Logistic model: RAB14; waist circumference; age; plasma glucose; HDL; ALT.	99.0% sensitivity 89.6% specificity 0.993 AUROC
[69]	Cirrhosis	67 cirrhosis 17 healthy	miR-571	0.910 AUROC
			miR-513-3p, miR-652	0.962 AUROC
			miR-513-3p, miR-652, miR-652	0.9676 AUROC
[70]	HCC	Meta-analysis of 20 studies (*n* = 1191) for HCC detection	Circulating tumor cell	60% sensitivity 95% specificity 0.91 AUROC
	HCC recurrence, mortality, tumor size	Meta-analysis of 18 studies (*n* = 1466) for HCC prognosis		3.03 (1.89–4.86) HR for recurrence 2.31 (1.55–3.42) HR for mortality 1.39 (1.15–1.66) RR for tumor size
[71]	HCC	Meta-analysis of 15 studies (*n* = 3686) for HCC detection	Circulating-free DNA	83% sensitivity 90% specificity 0.93 AUROC

MASLD: metabolic dysfunction–associated steatotic liver disease; PLS-DA: partial least squares discriminant analysis; AUROC: area under receiver operating characteristic; PDGFRβ: Platelet-Derived Growth Factor Receptor Beta; MASH: metabolic dysfunction-associated steatohepatitis; DM: diabetes mellitus; TG: triglyceride; ALT: alanine transaminase; HDL: high-density lipoprotein; HCC: hepatocellular carcinoma; HR: hazard ratio; RR: risk ratio.

## Data Availability

No new data were created or analyzed in this study. Data sharing is not applicable to this article.
